# Supplementary material for: Deep quantum neural networks on a superconducting processor
Source: Nat Commun. 2023 Jul 6;14:4006. doi: 10.1038/s41467-023-39785-8 (PMC10325994; doi:10.1038/s41467-023-39785-8)
Supplement: Supplementary file 1 — Supplementary Information [file 41467_2023_39785_MOESM1_ESM.pdf]

Supplementary Information: Deep quantum neural networks on a superconducting processor

Xiaoxuan Pan,[1,*] Zhide Lu,[1,*] Weiting Wang,[1] Ziyue Hua,[1] Yifang Xu,[1] Weikang Li,[1] Weizhou Cai,[1] Xuegang Li,[1] Haiyan Wang,[1] Yi-Pu Song,[1] Chang-Ling Zou,[2] Dong-Ling Deng,[1,3,†] and Luyan Sun[1,‡]

[1]*Center for Quantum Information, Institute for Interdisciplinary Information Sciences, Tsinghua University, Beijing 100084, China*
[2]*CAS Key Laboratory of Quantum Information, University of Science and Technology of China, Hefei, Anhui 230026, China*
[3]*Shanghai Qi Zhi Institute, No. 701 Yunjin Road, Xuhui District, Shanghai 200232, China*

SUPPLEMENTARY NOTE 1: THEORETICAL DETAILS FOR DEEP QUANTUM NEURAL NETWORKS

In classical machine learning, deep neural networks are characterized by the ability to extract high-level features from data. With the rapid development in quantum machine learning [1–5], we expect a quantum generalization of a deep neural network architecture to bring promising insights. Recently, a deep quantum neural network (DQNN) and a quantum analog of the backpropagation (BP) algorithm have been proposed [6]. In this ansatz, the quantum analog of a perceptron is a unitary operation which acts on qubits in two adjacent layers. During the training process, the unitary operator of a quantum perceptron is updated by multiplying the corresponding updating matrix.

In this paper, we experimentally demonstrate the training of parameterized DQNNs with a superconducting quantum processor. Equipped with the BP algorithm, we can efficiently calculate the gradients during the training process. Our scheme is feasible for the experimental implementation in the noisy intermediate scale quantum era. In this section, we will introduce the basic structures, optimization strategies, and training procedures for DQNNs.

Basic structures

As mentioned in the main text, our DQNNs have layer-by-layer structures, and qubits in two adjacent layers are connected with the quantum perceptrons. In our ansatz, the quantum perceptrons are engineered as parameterized quantum circuits. For simplicity in this paper, we consider that each quantum perceptron acts on only two qubits in two adjacent layers. The circuit structure of a quantum perceptron is composed of two single-qubit rotation gates $R_x(\theta_1)$ and $R_x(\theta_2)$ with θ_1 and θ_2 as the variational parameters, followed by a fixed two-qubit controlled-phase gate, which is shown in Fig. 1(b) in the main text. A sequential combination of the quantum perceptrons constitutes the layer-by-layer transition mapping between adjacent layers. In this way, the DQNN maps the information layerwise from the input layer to the output layer through hidden layers.

Now we consider a DQNN including L hidden layers. The total number of qubits in layer l is denoted as m_l. The unitary of a quantum perceptron which acts on the i-th qubit at layer $l-1$ and the j-th qubit at layer l is written as $U^l_{(i,j)}(\theta^l_{(i,j),1}, \theta^l_{(i,j),2})$, where $\theta^l_{(i,j),k}$ $(k = 1, 2)$ denote the variational parameters of the two R_x gates in the quantum perceptron $U^l_{(i,j)}$. The unitary product of all quantum perceptrons acting on the qubits in layers $l-1$ and l is denoted as:

$$U^l = \prod_{j=m_l}^{1} \prod_{i=m_{l-1}}^{1} U^l_{(i,j)}. \quad (1)$$

We note that qubits in layer l are initialized to a fiducial product state $|0\cdots0\rangle$, and then the quantum state ρ^l of the qubits in layer l can be written as the layer-by-layer transition mapping on ρ^{l-1}:

$$\rho^l = \mathcal{E}^l\left(\rho^{l-1}\right) \equiv \mathrm{tr}_{l-1}\left(U^l\left(\rho^{l-1} \otimes |0\cdots0\rangle_l\langle 0\cdots0|\right)U^{l^\dagger}\right). \quad (2)$$

In this way, the output state ρ^{out} can be expressed as a series of maps on ρ^{in}:

$$\rho^{\text{out}} = \mathcal{E}^{\text{out}}\left(\mathcal{E}^L\left(\ldots\mathcal{E}^2\left(\mathcal{E}^1\left(\rho^{\text{in}}\right)\right)\ldots\right)\right). \quad (3)$$

Optimization strategies

With the basic structures discussed above, now we can specify the learning tasks. In this paper, we consider two machine learning tasks. The first task is learning a target quantum channel. We expect the output state given by the DQNN to be as close

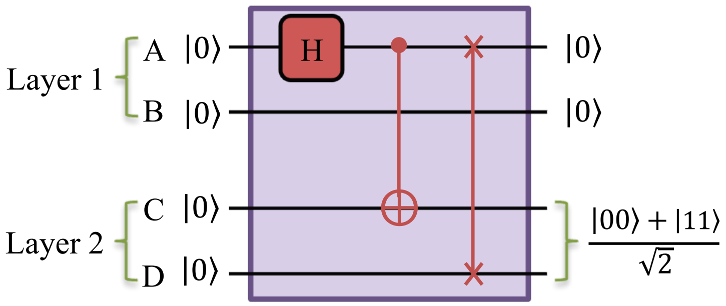

Supplementary Figure 1. **An example of producing an entangled state without intra-layer gates.** Each qubit is initialized in the $|0\rangle$ state. First, a Hadamard gate acts on qubit A, then a CNOT gate acts on qubits A and C, where A is the control qubit. Finally, a SWAP gate acts on qubits A and D. The produced output state is an entangled state $(|00\rangle + |11\rangle)/\sqrt{2}$.

as possible to the output state given by the target quantum channel for each input state. We aim to maximize the mean fidelity between output states given by the DQNN (ρ_x^{out}) and the target quantum channel (τ_x^{out}) averaged over N training data:

$$F = \frac{1}{N}\sum_{x=1}^{N} F_x(\rho_x^{\text{out}}, \tau_x^{\text{out}}) = \frac{1}{N}\sum_{x=1}^{N}\left[\text{tr}\sqrt{\sqrt{\tau_x^{\text{out}}}\rho_x^{\text{out}}\sqrt{\tau_x^{\text{out}}}}\right]. \tag{4}$$

Here, we use $\left[\text{tr}\sqrt{\sqrt{\tau_x^{\text{out}}}\rho_x^{\text{out}}\sqrt{\tau_x^{\text{out}}}}\right]$ as the fidelity between mixed quantum states. When the target quantum channel is a quantum unitary and ρ_x^{in} are pure states, τ_x^{out} will also be pure states that can be written as $|\phi_x^{\text{out}}\rangle\langle\phi_x^{\text{out}}|$. Then the mean fidelity can be simplified to be:

$$F = \frac{1}{N}\sum_{x=1}^{N} \text{tr}(\rho_x^{\text{out}}|\phi_x^{\text{out}}\rangle\langle\phi_x^{\text{out}}|). \tag{5}$$

Another way to learn the target quantum channel is to minimize the cost function given by :

$$C = \frac{1}{N}\sum_{x=1}^{N} C_x(\rho_x^{\text{out}}, \tau_x^{\text{out}}) = \frac{1}{N}\sum_{x=1}^{N}\|\rho_x^{\text{out}} - \tau_x^{\text{out}}\|_F^2 = \frac{1}{N}\sum_{x=1}^{N}\text{tr}[(\rho_x^{\text{out}} - \tau_x^{\text{out}})^2], \tag{6}$$

where $\|\cdot\|_F$ denotes the Frobenius norm.

The second task is learning the ground state energy of a Hamiltonian H. We aim to minimize the energy estimate $\bar{E}$ of this Hamiltonian computed with the DQNN output state ρ^{out}: $\bar{E} = \text{tr}\,(\rho^{\text{out}}H)$. We note two important points. First, the ground state of the molecular hydrogen is a pure quantum state, and DQNNs generally produce mixed states in the output layer. So in the training process to approximate the ground state, the qubits in the output layer will be gradually disentangled from the other qubits. Second, although there are no intra-layer gates in our DQNNs, we can still obtain entangled pure states in the output layer via the interaction with the extra layers (a specific example is given in Supplementary Fig. 1).

To maximize the mean fidelity or minimize the energy estimate, we adapt the gradient descent method. In the main text, we mention that our DQNNs with the layer-by-layer architecture allow the quantum BP algorithm. Via this algorithm, one only requires the information from two adjacent layers to calculate the gradients with respect to all gate parameters at these two layers. In other words, the derivative of the mean fidelity or the energy estimate with respect to $\theta_{(i,j),k}^l$ can be written as $G(\boldsymbol{\theta^l}, \rho^{l-1}, \sigma^l)$, where $\boldsymbol{\theta^l}$ incorporates all gate parameters in layers $l-1$ and l.

Here, we derive the formula for $G(\boldsymbol{\theta^l}, \rho^{l-1}, \sigma^l)$. We first consider a function f with the form $f(\rho^{\text{out}}, X) = \text{tr}(X\rho^{\text{out}})$, where X is a Hermitian matrix related to specific tasks. The derivative of f with respect to $\theta_{(i,j),k}^l$ can be expressed as:

$$\begin{aligned}\frac{\partial f(\rho^{\text{out}}, X)}{\partial\theta_{(i,j),k}^l} &= G(\boldsymbol{\theta^l}, \rho^{l-1}, \sigma^l)\\ &= \text{tr}\left(\frac{\partial U^l}{\partial\theta_{(i,j),k}^l}\left(\rho^{l-1}\otimes|0\rangle_l\langle 0|\right)U^{l^\dagger}\left(\mathbb{I}_{l-1}\otimes\sigma^l\right)\right) + \text{h.c.},\end{aligned} \tag{7}$$

where $\mathrm{h.c.}$ stands for the Hermitian conjugate of the preceding terms. We show the proof as follows:

Proof.

$$\begin{aligned}
\frac{\partial f(\rho^{\text{out}},X)}{\partial\theta^l_{(i,j),k}} &= G(\boldsymbol{\theta^l},\rho^{l-1},\sigma^l) = \mathrm{tr}\left(\frac{\partial\rho^{\text{out}}}{\partial\theta^l_{(i,j),k}}X\right)\\
&= \mathrm{tr}\left(\left(\mathrm{tr}_{\text{in,hidden}}\left(U^{\text{out}}\dots U^{l+1}\frac{\partial U^l}{\partial\theta^l_{(i,j),k}}U^{l-1}\dots U^1\rho^{\text{in}}\otimes|0\cdots0\rangle_{\text{hid,out}}\langle0\cdots0|\mathcal{U}^\dagger\right)+\mathrm{h.c.}\right)X\right)\\
&= \mathrm{tr}\left(\left(U^{\text{out}}\dots U^{l+1}\frac{\partial U^l}{\partial\theta^l_{(i,j),k}}U^{l-1}\dots U^1\rho^{\text{in}}\otimes|0\cdots0\rangle_{\text{hid,out}}\langle0\cdots0|\mathcal{U}^\dagger\right)\cdot\left(\mathbb{I}_{\text{in,hidden}}\otimes X\right)\right)+\mathrm{h.c.}\\
&= \mathrm{tr}\left(\frac{\partial U^l}{\partial\theta^l_{(i,j),k}}U^{(l-1)}\dots U^1\rho^{\text{in}}\otimes|0\cdots0\rangle_{\text{hid,out}}\langle0\cdots0|U^{1^\dagger}\dots U^{(l-1)^\dagger}\right.\\
&\qquad\left.U^{(l)^\dagger}U^{(l+1)^\dagger}\dots U^{\text{out}^\dagger}\left(\mathbb{I}_{\text{in,hidden}}\otimes X\right)U^{\text{out}}\dots U^{(l+1)}\right)+\mathrm{h.c.}\\
&= \mathrm{tr}\left(\frac{\partial U^l}{\partial\theta^l_{(i,j),k}}\left(\mathrm{tr}_{l,\dots\text{out}}(T_1)\otimes|0\cdots0\rangle_{l,\dots,\text{out}}\langle0\cdots0|\right)U^{l^\dagger}\left(\mathbb{I}_{0,\dots,l-1}\otimes\mathrm{tr}_{0,\dots l-1}(T_2)\right)\right)+\mathrm{h.c.}\\
&= \mathrm{tr}\left(\left(\left(\frac{\partial U^l}{\partial\theta^l_{(i,j),k}}\left(\mathrm{tr}_{l,\dots\text{out}}(T_1)\otimes|0\rangle_l\langle0|\right)U^{l^\dagger}\right)\otimes\mathbb{I}_{l+1,\dots\text{out}}\right)\right.\\
&\qquad\left.\left(\mathbb{I}_{0,\dots l}\otimes|0\rangle_{l+1,\dots\text{out}}\langle0|\right)\left(\mathbb{I}_{0,\dots l-1}\otimes\mathrm{tr}_{0,\dots l-1}(T_2)\right)\right)+\mathrm{h.c.}\\
&= \mathrm{tr}\left(\left(\left(\frac{\partial U^l}{\partial\theta^l_{(i,j),k}}\left(\rho^{l-1}\otimes|0\rangle_l\langle0|\right)U^{l^\dagger}\right)\otimes\mathbb{I}_{l+1,\dots\text{out}}\right)\left(\mathbb{I}_{l-1,l}\otimes|0\rangle_{l+1,\dots\text{out}}\langle0|\right)\left(\mathbb{I}_{l-1}\otimes\mathrm{tr}_{0,\dots l-1}(T_2)\right)\right)+\mathrm{h.c.}\\
&= \mathrm{tr}\left(\frac{\partial U^l}{\partial\theta^l_{(i,j),k}}\left(\rho^{l-1}\otimes|0\rangle_l\langle0|\right)U^{l^\dagger}\left(\mathbb{I}_{l-1}\otimes\sigma^l\right)\right)+\mathrm{h.c.},
\end{aligned}\tag{8}$$

where $\mathcal{U}\equiv U^{\text{out}}U^LU^{L-1}\dots U^1$. We use the shorthands $T_1=U^{(l-1)}\dots U^1\rho^{\text{in}}\otimes|0\cdots0\rangle_{\text{hid,out}}\langle0\cdots0|U^{1^\dagger}\dots U^{(l-1)^\dagger}$, and $T_2=1/(2^{\sum_{i=m_0}^{m_{l-1}}})U^{(l+1)^\dagger}\dots U^{\text{out}^\dagger}\left(\mathbb{I}_{\text{in,hidden}}\otimes X\right)U^{\text{out}}\dots U^{(l+1)}$. We define $\rho^{l-1}=\mathrm{tr}_{1,\dots,l-2,l,\dots,\text{out}}(T_1)$ as the quantum states of the qubits in layer $l-1$ in the forward process, and $\sigma^l=\mathrm{tr}_{l+1,\dots,\text{out}}\left(\left(\mathbb{I}_l\otimes|0\cdots0\rangle_{l+1,\dots,\text{out}}\langle0\cdots0|\right)\cdot\mathrm{tr}_{1,\dots,l-1}(T_2)\right)$ as the backward term in layer l. From this formula we obtain the recursive relation between σ^{l-1} and σ^l:

$$\sigma^{l-1}=\mathcal{F}^l(\sigma^l)=\mathrm{tr}_l\left(\left(\mathbb{I}_{l-1}\otimes|0\rangle_l\langle0|\right)U^{l^\dagger}\left(\mathbb{I}_{l-1}\otimes\sigma^l\right)U^l\right),\tag{9}$$

where $\mathcal{F}^l$ is the adjoint channel of $\mathcal{E}^l$, and $\sigma^{\text{out}}=X$. From this recursive relation, we can obtain the backward terms layerwise from the output layer to the input layer in the backward process.

Specially, if the gate with parameter $\theta^l_{(i,j),k}$ in the DQNN is of the form $e^{-\frac{i}{2}\theta^l_{(i,j),k}P_n}$ with P_n belonging to the Pauli group, we can utilize the "parameter shift rule" to calculate the gradient of f:

$$\frac{\partial f(\rho,\sigma)}{\partial\theta^l_{(i,j),k}}=G(\boldsymbol{\theta^l},\rho^{l-1},\sigma^l)=\frac{1}{2}(h_+-h_-),\tag{10}$$

where $h_\pm=\mathrm{tr}(\rho^l_\pm\sigma^l)=\mathrm{tr}\left(U^l_\pm\left(\rho^{l-1}\otimes|0\rangle_l\langle0|\right)U^{l^\dagger}_\pm\left(\mathbb{I}_{l-1}\otimes\sigma^l\right)\right)$, $\rho^l_\pm=\mathrm{tr}_{l-1}\left(U^l_\pm\left(\rho^{l-1}\otimes|0\rangle_l\langle0|\right)U^{l^\dagger}_\pm\right)$, and $U^l_\pm$ denotes the unitary that replaces the parameter $\theta^l_{(i,j),k}$ in U^l with $\theta^l_{(i,j),k}\pm\frac{\pi}{2}$. We show the proof as follows:

Proof.

$$
\begin{aligned}
2\cdot\frac{\partial f(\rho^{\text{out}},X)}{\partial\theta^l_{(i,j),k}} &= 2\cdot G(\boldsymbol{\theta^l},\rho^{l-1},\sigma^l) = 2\cdot\operatorname{tr}\left(\frac{\partial\rho^{\text{out}}}{\partial\theta^l_{(i,j),k}}X\right)\\
&= \operatorname{tr}\left(\left(\operatorname{tr}_{\text{in,hidden}}\left(U^{\text{out}}\dots U^{l+1}U^l_+U^{l-1}\dots U^1\rho^{\text{in}}\otimes|0\cdots0\rangle_{\text{hid,out}}\langle0\cdots0|U^{1^\dagger}\cdots U^{l^\dagger}_+\cdots U^{\text{out}^\dagger}\right)\right)X\right)\\
&- \operatorname{tr}\left(\left(\operatorname{tr}_{\text{in,hidden}}\left(U^{\text{out}}\dots U^{l+1}U^l_-U^{l-1}\dots U^1\rho^{\text{in}}\otimes|0\cdots0\rangle_{\text{hid,out}}\langle0\cdots0|U^{1^\dagger}\cdots U^{l^\dagger}_-\cdots U^{\text{out}^\dagger}\right)\right)X\right).
\end{aligned}
\tag{11}
$$

Now we prove the first term equals to h_+. In the same way, we can prove the second term equals to h_-. The first term can be written as:

$$
\begin{aligned}
&\operatorname{tr}\left(\left(\operatorname{tr}_{\text{in,hidden}}\left(U^{\text{out}}\dots U^{l+1}U^l_+U^{l-1}\dots U^1\rho^{\text{in}}\otimes|0\cdots0\rangle_{\text{hid,out}}\langle0\cdots0|U^{1^\dagger}\cdots U^{l^\dagger}_+\cdots U^{\text{out}^\dagger}\right)\right)X\right)\\
&=\operatorname{tr}\left(\left(U^{\text{out}}\dots U^{l+1}U^l_+U^{l-1}\dots U^1\rho^{\text{in}}\otimes|0\cdots0\rangle_{\text{hid,out}}\langle0\cdots0|U^{1^\dagger}\cdots U^{l^\dagger}_+\cdots U^{\text{out}^\dagger}\right)\left(\mathbb{I}_{\text{in,hidden}}\otimes X\right)\right)\\
&=\operatorname{tr}\Big(U^l_+U^{(l-1)}\dots U^1\rho^{\text{in}}\otimes|0\cdots0\rangle_{\text{hid,out}}\langle0\cdots0|U^{1^\dagger}\dots U^{(l-1)^\dagger}U^{l^\dagger}_+U^{(l+1)^\dagger}\dots U^{\text{out}^\dagger}\\
&\qquad\left(\mathbb{I}_{\text{in,hidden}}\otimes X\right)U^{\text{out}}\dots U^{(l+1)}\Big)\\
&=\operatorname{tr}\left(U^l_+\left(\operatorname{tr}_{l,\dots\text{out}}(T_1)\otimes|0\cdots0\rangle_{l,\dots\text{out}}\langle0\cdots0|\right)U^{l^\dagger}_+\left(\mathbb{I}_{0,\dots l-1}\otimes\operatorname{tr}_{0,\dots l-1}(T_2)\right)\right)\\
&=\operatorname{tr}\Big(\left(\left(U^l_+\left(\operatorname{tr}_{l,\dots\text{out}}(T_1)\otimes|0\rangle_l\langle0|\right)U^{l^\dagger}_+\right)\otimes\mathbb{I}_{l+1,\dots\text{out}}\right)\left(\mathbb{I}_{0,\dots l}\otimes|0\rangle_{l+1,\dots\text{out}}\langle0|\right)\\
&\qquad\left(\mathbb{I}_{0,\dots l-1}\otimes\operatorname{tr}_{1,\dots l-1}(T_2)\right)\Big)\\
&=\operatorname{tr}\left(\left(\left(U^l_+\left(\rho_{l-1}\otimes|0\rangle_l\langle0|\right)U^{l^\dagger}_+\right)\otimes\mathbb{I}_{l+1,\dots\text{out}}\right)\left(\mathbb{I}_{l-1,l}\otimes|0\rangle_{l+1,\dots\text{out}}\langle0|\right)\left(\mathbb{I}_{l-1}\otimes\operatorname{tr}_{0,\dots,l-1}(T_2)\right)\right)\\
&=\operatorname{tr}\left(U^l_+\left(\rho^{l-1}\otimes|0\rangle_l\langle0|\right)U^{l^\dagger}_+\left(\mathbb{I}_{l-1}\otimes\sigma^l\right)\right)\\
&=\operatorname{tr}(\rho^l_+\sigma^l)\\
&=h_+.
\end{aligned}
\tag{12}
$$

With the gradients of f obtained above, we can derive the gradients of the mean fidelity F (or the cost function C) and the energy estimate $\bar{E}$ in the two tasks that are discussed in the main text.

For the task of learning a target quantum channel, we can maximize the mean fidelity F (Supplementary Eq. 4) or minimize the cost function C (Supplementary Eq. 6). When we aim to maximize the mean fidelity F, for each input state, we consider the derivative of F_x with respect to $\theta^l_{(i,j),k}$. For convenience, we omit the superscript and subscript of F_x, ρ^{out}_x, and τ^{out}_x, and use the shorthand $A=\tau^{1/2}\rho\tau^{1/2}$, $B=\sqrt{A}$, then

$$
\frac{\partial F}{\partial\theta^l_{(i,j),k}}=\operatorname{tr}\left(\frac{\partial B}{\partial\theta^l_{(i,j),k}}\right).
\tag{13}
$$

Now, we further omit the superscript and subscript of $\theta^l_{(i,j),k}$.

$$
\frac{\partial A}{\partial\theta}=\frac{\partial\left(B^2\right)}{\partial\theta}=B\cdot\frac{\partial B}{\partial\theta}+\frac{\partial B}{\partial\theta}\cdot B\Rightarrow\frac{\partial A}{\partial\theta}\cdot B^{-1}=B\cdot\frac{\partial B}{\partial\theta}B^{-1}+\frac{\partial B}{\partial\theta},
\tag{14}
$$

hence,

$$
\operatorname{tr}\left(\frac{\partial A}{\partial\theta}\cdot B^{-1}\right)=\operatorname{tr}\left(B\cdot\frac{\partial B}{\partial\theta}B^{-1}\right)+\operatorname{tr}\left(\frac{\partial B}{\partial\theta}\right)=\operatorname{tr}\left(\frac{\partial B}{\partial\theta}B^{-1}B\right)+\operatorname{tr}\left(\frac{\partial B}{\partial\theta}\right)=2\operatorname{tr}\left(\frac{\partial B}{\partial\theta}\right).
\tag{15}
$$

This yields

$$\frac{\partial F}{\partial \theta}=\frac{1}{2}\operatorname{tr}\left(\frac{\partial A}{\partial \theta}\cdot B^{-1}\right)=\frac{1}{2}\operatorname{tr}\left(\tau^{1/2}\frac{\partial \rho}{\partial \theta}\tau^{1/2}\cdot B^{-1}\right)=\frac{1}{2}\operatorname{tr}\left(\frac{\partial \rho}{\partial \theta}\cdot \tau^{1/2}B^{-1}\tau^{1/2}\right), \tag{16}$$

which has the same form as the derivative of $\operatorname{tr}(\rho^{\text{out}}X)$ with $\tau^{1/2}B^{-1}\tau^{1/2}$ analogous to X. When the target quantum channel is a unitary, the fidelity can be simplified to Supplementary Eq. 5, which has the same form as $\operatorname{tr}(\rho^{\text{out}}X)$ with $\rho^{\text{out}}=|\phi_x^{\text{out}}\rangle\langle\phi_x^{\text{out}}|$ being analogous to X.

When we aim to minimize the cost function in Supplementary Eq. 6, for each input state, we consider the derivative of C_x with respect to $\theta^l_{(i,j),k}$. For convenience, we omit the superscript and subscript of C_x, ρ_x^{out}, τ_x^{out}, and $\theta^l_{(i,j),k}$, then

$$\frac{\partial C}{\partial \theta}=2\operatorname{tr}\left[(\rho-\tau)\frac{\partial \rho}{\partial \theta}\right]=2\operatorname{tr}\left(\rho\frac{\partial \rho}{\partial \theta}\right)-2\operatorname{tr}\left(\tau\frac{\partial \rho}{\partial \theta}\right), \tag{17}$$

where $(\rho-\tau)$ is analogous to X.

In the task of learning the ground state energy of a Hamiltonian H, the energy estimate $\operatorname{tr}(\rho^{\text{out}}H)$ has the same form as $\operatorname{tr}(\rho^{\text{out}}X)$, where H is analogous to X. So we can derive the gradients of the energy estimate $\bar{E}$ according to $G(\boldsymbol{\theta^l},\rho^{l-1},\sigma^l)$. With the gradients obtained, we can update the variational parameters in the DQNN by gradient descent methods.

Training procedures

In this section, we give a detailed description of how our DQNNs are trained via the quantum BP algorithm for different tasks.

For the task of learning a quantum channel, first we need to generate the training dataset. Here, we randomly choose parameters $\boldsymbol{\theta_t}$ in the DQNN to generate a specific target quantum channel that we aim to learn. Then we apply the target quantum channel on each input state to obtain the corresponding output state to constitute the training dataset $\{(\rho_x^{\text{in}},\tau_x^{\text{out}})\}_{x=1}^N$ with N being the size of the training dataset. We assume the DQNN used in this task includes L hidden layers with a total number m_l of qubits in layer l.

In the following, we discuss the strategy to experimentally implement the training. In the forward process, we perform the forward channel to obtain $\rho_x^1,\rho_x^2,\ldots,\rho_x^{\text{out}}$. To evaluate the gradient with respect to $\theta^l_{(i,j),k}$ for each training data, we also need to obtain $\rho^l_{\pm,x}$. In the backward process, we perform the backward channel to obtain $\sigma_x^L,\sigma_x^{L-1},\ldots,\sigma_x^1$. The gradient with respect to $\theta^l_{(i,j),k}$ for each training data can be evaluated with Supplementary Eq. 10. The training procedure is described as follows:

1. Initialization:
   Randomly choose initial gate parameters for all perceptrons in the DQNN, which is denoted as $\boldsymbol{\theta_I}$.

2. Forward process:
   For each training data $\{(\rho_x^{\text{in}},\tau_x^{\text{out}})\}$, apply forward channels $\mathcal{E}^1$, $\mathcal{E}^2$,. . ., $\mathcal{E}^{\text{out}}$ on ρ_x^{in} to obtain $\rho_x^1,\rho_x^2,\ldots,\rho_x^{\text{out}}$ successively. For each parameter $\theta^l_{(i,j),k}$, we need to obtain $\rho^l_{\pm,x}$, where $\rho^l_{\pm,x}=\operatorname{tr}_{l-1}\left(U^l_\pm\left(\rho^{l-1}\otimes|0\rangle_l\langle 0|\right)U^{l\dagger}_\pm\right)$.

   Forward channel $\mathcal{E}^l$: According to the main text, the forward channel $\mathcal{E}^l$ applies on qubits in layer $l-1$ of the quantum state ρ_x^{l-1}, and produces ρ_x^l in layer l according to $\rho_x^l=\mathcal{E}^l\left(\rho_x^{l-1}\right)=\operatorname{tr}_{l-1}\left(U^l\left(\rho_x^{l-1}\otimes|0\cdots0\rangle_l\langle0\cdots0|\right)U^{l^\dagger}\right)$. In our experiment, we prepare m_l qubits in layer l to the fiducial product state $|0\cdots0\rangle$ at first. Then we apply all quantum perceptrons acting on qubits in layers $l-1$ and l. $\rho^l_{\pm,x}$ is obtained by applying $U^l_\pm$ that replaces the parameter $\theta^l_{(i,j),k}$ in U^l with $\theta^l_{(i,j),k}\pm\frac{\pi}{2}$. $\rho^l_{\pm,x}$ are extracted by quantum state tomography.

3. Backward process:
   There are two approaches. For the first approach, we aim to maximize the mean fidelity F (see Supplementary Eq. 4). For each training data $\{(\rho_x^{\text{in}},\tau_x^{\text{out}})\}$, we classically calculate $\sigma_x^{\text{out}}=(\tau_x^{\text{out}})^{1/2}((\tau_x^{\text{out}})^{1/2}\rho_x^{\text{out}}(\tau_x^{\text{out}})^{1/2})^{-1/2}(\tau_x^{\text{out}})^{1/2}$ and prepare σ_x^{out}, and then apply backward channels $\mathcal{F}^{\text{out}}$, $\mathcal{F}^L$,. . ., $\mathcal{F}^1$ on σ_x^{out} to successively obtain $\sigma_x^L,\sigma_x^{L-1},\ldots,\sigma_x^0$. In this case, we need to prepare the general mixed state σ_x^{out}, which will be challenging in experiment when the network scales up in the width of the output layer.
   For the second approach, we aim to minimize the cost function C (see Supplementary Eq. 6). We prepare $\sigma_x^{\text{out},1}=\rho_x^{\text{out}}$and $\sigma_x^{\text{out},2}=\tau_x^{\text{out}}$, and then apply backward channels on them to obtain their corresponding backward terms (the backward terms for $\sigma_x^{\text{out},1}$ and $\sigma_x^{\text{out},2}$ are separately used to evaluate the first and second terms in Supplementary Eq. 17). In this case, we need to prepare ρ_x^{out} and τ_x^{out}, which can be directly obtained in experiment by running the DQNN and the target

quantum channel.

Backward channel $\mathcal{F}^l$: The backward channel $\mathcal{F}^l$ applies on backward term σ^l and produces σ^{l-1} according to $\sigma^{l-1} = \mathcal{F}^l(\sigma^l) = \mathrm{tr}_l\left((\mathbb{I}_{l-1} \otimes |0\rangle_l\langle 0|)\, U^{l^\dagger}\, (\mathbb{I}_{l-1} \otimes \sigma^l)\, U^l\right)$. Here, we provide a possible experimental proposal to realize $\mathcal{F}^l$ in the future. In experiment, the qubits in layer $l-1$ are prepared to be a maximally mixed state. This can be done in several ways. For example, we can take another m_{l-1} ancillary qubits and prepare an entangled pure state $\sum_{q_A=q_B} \frac{1}{\sqrt{2^{m_{l-1}}}} |q_A, q_B\rangle$, where the summation goes over all of the computational bases in subsystem A with m_{l-1} qubits. After tracing out the ancillary subsystem B, the m_{l-1}-qubit subsystem A is in a maximally mixed state. Then, the inverse of U^l acts on the state $(\mathbb{I}_{l-1}/2^{m_{l-1}}) \otimes \sigma^l$. Next, the qubits in layer l are measured in the Z basis, and σ^{l-1} will be obtained when the measurement outcome of the qubits in layer l is $|0\rangle_l$. σ^{l-1} can be extracted by quantum state tomography. Finally, σ^{l-1} should be multiplied by a factor $2^{m_{l-1}} \times \langle 0|_l\, \mathrm{tr}_{l-1}(U^{l^\dagger}\left[(\mathbb{I}_{l-1}/2^{m_{l-1}}) \otimes \sigma^l\right] U^l)|0\rangle_l$, where $\langle 0|_l\, \mathrm{tr}_{l-1}(U^{l^\dagger}\left[(\mathbb{I}_{l-1}/2^{m_{l-1}}) \otimes \sigma^l\right] U^l)|0\rangle_l$ is obtained by measuring qubits in layer l in the Z basis and calculating the probability of measurement outcome being $|0\rangle_l$. The required number of measurements, scaling with the dimension of the Hilbert space of qubits in layer l, is $O(2^{m_l})$.

4. Evaluate the mean fidelity (or the cost function) and the gradients:
Compute the mean fidelity (Supplementary Eq. 4) or the cost function (Supplementary Eq. 6). Calculate the gradient of the mean fidelity (or the cost function) with respect to $\theta^l_{(i,j),k}$ for each training data according to Supplementary Eq. 10, and then take the average over the whole training dataset. Finally, update each $\theta^l_{(i,j),k}$ with the learning rate ϵ according to:

$$\theta^l_{(i,j),k} \to \theta^l_{(i,j),k} + \epsilon * \frac{1}{N} \sum_{x=1}^{N} G(\boldsymbol{\theta^l}, \rho_x^{l-1}, \sigma_x^l). \tag{18}$$

5. Repeat 2, 3 and 4 for s_0 steps.

In the experimental proposal to implement the backward channel, additional ancillary qubits are required for preparing maximally mixed states. In addition, we need to separately evaluate two terms in Supplementary Eq. 17. So the required experimental accuracy is more stringent than that for implementing the forward channel. In our current work, we experimentally perform the forward process on the quantum processor while implementing the backward channel on a classical computer. Our algorithm can be summarized as the pseudocode in Algorithm 1.

In fact, we can use the SWAP test to estimate the value of $h_\pm = \mathrm{tr}(\rho^l_\pm \sigma^l)$ in Supplementary Eq. 17. For two mixed states $\rho^l_\pm$ and σ^l, the probability of passing the SWAP test is $[1 + \mathrm{tr}(\rho^l_\pm \sigma^l)]/2$ [7, 8]. In this way, we can directly evaluate the gradient without doing state tomography to obtain $\rho^l_\pm$ and σ^l. However, performing the SWAP test requires experimental realization of the controlled-SWAP gate with high fidelity, which is challenging in current experiments when the controlled-SWAP gate acts on multiple qubits.

Algorithm 1 Training the DQNN for learning quantum channels via the quantum backpropagation algorithm

Input The DQNN model with L hidden layers, initial parameters $\boldsymbol{\theta_I}$, input quantum states $\{(\rho_x^{\text{in}})\}_{x=1}^N$, iteration steps s_0 , learning rate ϵ,
Output The trained DQNN
Generate the training dataset: choose parameters $\boldsymbol{\theta_t}$ for the DQNN, which serves as the target quantum channel, and then apply it to each input state to obtain the corresponding output state, which constitute the training dataset $\{(\rho_x^{\text{in}}, \tau_x^{\text{out}})\}_{x=1}^N$.
for $s = 1$ to s_0 **do**
Forward: for each training data $\{(\rho_x^{\text{in}}, \tau_x^{\text{out}})\}$, apply forward channels $\mathcal{E}^1, \mathcal{E}^2, \ldots, \mathcal{E}^{\text{out}}$ on ρ_x^{in} to obtain $\rho_x^1, \rho_x^2, \ldots, \rho_x^{\text{out}}$ successively .
Backward: for each training data $\{(\rho_x^{\text{in}}, \tau_x^{\text{out}})\}$, calculate $\sigma^{\text{out}} = (\tau_x^{\text{out}})^{1/2}((\tau_x^{\text{out}})^{1/2}\rho_x^{\text{out}}(\tau_x^{\text{out}})^{1/2})^{-1/2}(\tau_x^{\text{out}})^{1/2}$, and then apply backward channels $\mathcal{F}^{\text{out}}, \mathcal{F}^L, \ldots, \mathcal{F}^1$ on σ^{out} to successively obtain $\sigma_x^L, \sigma_x^{L-1}, \ldots, \sigma_x^0$.
Gradients: calculate the gradient with respect to $\theta^l_{(i,j),k}$ for each training data: $\frac{\partial F_x(\rho_x^{\text{out}}, \tau_x^{\text{out}})}{\partial \theta^l_{(i,j),k}} = G(\boldsymbol{\theta^l}, \rho_x^{l-1}, \sigma_x^l)$, and then take the average over the whole training dataset: $\frac{1}{N}\sum_{x=1}^N G(\boldsymbol{\theta^l}, \rho_x^{l-1}, \sigma_x^l)$.
Update: update each $\theta^l_{(i,j),k}$ according to $\theta^l_{(i,j),k} \to \theta^l_{(i,j),k} + \epsilon * \frac{1}{N}\sum_{x=1}^N G(\boldsymbol{\theta^l}, \rho_x^{l-1}, \sigma_x^l)$.
end for
Output the trained DQNN

For the task of learning the ground state energy of a Hamiltonian H, we provide the pseudocode in Algorithm 2.

Algorithm 2 Training the DQNN for learning the ground state for some Hamiltonian via the quantum backpropagation algorithm

Input The DQNN model with L hidden layers, initial parameters $\boldsymbol{\theta_I}$, Hamiltonian H, iteration steps s_0 , learning rate ϵ.
Output The trained DQNN
for $s = 1$ to s_0 **do**
  Forward: apply forward channels $\mathcal{E}^1$, $\mathcal{E}^2$,. . ., $\mathcal{E}^{\text{out}}$ on initial fiducial product state $|0 \cdots 0\rangle$ to obtain $\rho^1, \rho^2, \ldots, \rho^{\text{out}}$ successively .
  Backward: apply backward channels $\mathcal{F}^{\text{out}}$, $\mathcal{F}^L$,. . ., $\mathcal{F}^1$ on $\sigma^{\text{out}} = H$ to successively obtain $\sigma^L, \sigma^{L-1}, \ldots, \sigma^0$.
  Gradients: calculate the gradient with respect to $\theta^l_{(i,j),k}$: $\frac{\partial \bar{E}(\rho^{\text{out}},H)}{\partial \theta^l_{(i,j),k}} = G(\boldsymbol{\theta^l}, \rho^{l-1}, \sigma^l)$.
  Update: update each $\theta^l_{(i,j),k}$ according to $\theta^l_{(i,j),k} \to \theta^l_{(i,j),k} - \epsilon * G(\boldsymbol{\theta^l}, \rho^{l-1}, \sigma^l)$.
end for
Output the trained DQNN

SUPPLEMENTARY NOTE 2: DISCUSSION ABOUT THE EFFICIENCY OF THE QUANTUM BP ALGORITHM

In this section, we discuss the efficiency of the quantum BP algorithm in training DQNNs in terms of the required number of copies for each training data per training iteration.

Consider the task of learning a target quantum channel. According to Supplementary Eq. 10, we need to carry out multiple measurements for quantum state tomography of $\rho^l_\pm$ and σ^l. As more measurements mean more copies of each training data are needed, we use the required number of copies as a measure of the training efficiency. This is a crucial parameter especially when producing the training data is expensive.

Here, we evaluate the required numbers of copies with and without the quantum BP algorithm. As mentioned above, we assume a DQNN including L hidden layers. The total number of qubits in layer l is denoted as m_l. Without the quantum BP algorithm, to calculate the derivative of the cost function with respect to each parameter θ in each round of training, we need to run the full DQNN separately with $\theta + \delta\theta$ and θ to obtain the corresponding output states and extract them by quantum state tomography. So the required number of copies for each training data $(\rho^{\text{in}}_x, \tau^{\text{out}}_x)$ in an iteration is:

$$\begin{aligned}
N_{\text{copies}} &\propto \sum_{l=0}^{L} (d^2_{\text{out}} - 1) \times n^{l,l+1}_{\text{params}} \\
&= (d^2_{\text{out}} - 1) \times n_{\text{params}} \\
&= \sum_{l=0}^{L} (4^{m_{\text{out}}} - 1) \times 2 \times m_l \times m_{l+1} \\
&= (4^{m_{\text{out}}} - 1) \times 2 \times m_L \times m_{\text{out}} + \sum_{l=0}^{L-1} (4^{m_{\text{out}}} - 1) \times 2 \times m_l \times m_{l+1},
\end{aligned} \tag{19}$$

where $d_{\text{out}} = 2^{m_{\text{out}}}$ is the dimension of the Hilbert space for the output layer, and $(d^2_{\text{out}} - 1)$ arises because it requires $O(d^2_{\text{out}} - 1)$ measurements to characterize a state on a d_{out}-dimensional Hilbert space; $n^{l,l+1}_{\text{params}} = 2 \times m_l \times m_{l+1}$ is the number of parameters between layers l and $l + 1$, and n_{params} is the total number of parameters in the DQNN given by:

$$n_{\text{params}} = \sum_{l=0}^{L} 2 \times m_l \times m_{l+1}. \tag{20}$$

For the training algorithm with the quantum BP algorithm (the backward channel is implemented according to the experimental proposal we mentioned above), to evaluate the derivative with respect to each parameter between layers $l - 1$ and l in Supplementary Eq. 10, we do tomography to find states $\rho^l_\pm$ and σ^l, and the number of copies of each pair required in a round is $O(d^2_l - 1)$. Additionally, we also estimate the number of copies required for realizing the backward channel to obtain σ^l. As discussed in the last section, to obtain σ^l, we need to successively apply the backward channel $\mathcal{F}^{\text{out}}$, $\mathcal{F}^L$,. . ., $\mathcal{F}^{l+1}$, which requires $O(2^{m_{\text{out}}+\ldots m_{l+1}})$ measurements. Then, the required number of copies of each training data in an iteration is:

$$\begin{aligned}
N^{\text{BP}}_{\text{copies}} &\propto (d^2_{\text{out}} - 1) \times n^{L,\text{out}}_{\text{params}} + \sum_{l=0}^{L-1} (d^2_{l+1} - 1) \times n^{l,l+1}_{\text{params}} \times 2^{m_{\text{out}}+\ldots m_{l+2}} \\
&= (4^{m_{\text{out}}} - 1) \times 2 \times m_L \times m_{\text{out}} + \sum_{l=0}^{L-1} (4^{m_{l+1}} - 1) \times 2 \times m_l \times m_{l+1} \times 2^{m_{\text{out}}+\ldots m_{l+2}}.
\end{aligned} \tag{21}$$

We find that, when the width of the hidden layers is much smaller than that of the output layer, it is possible to have $N_{\text{copies}}^{\text{BP}} < N_{\text{copies}}$. We consider a simple case, assuming $m_0 = m_{\text{out}} = M$, $m_1 = m_2 = \dots m_L = m$ (all hidden layers have the same width), and $M > (L+1) \times m$. In this case, as

$$\underbrace{(4^{m_{l+1}} - 1) \times 2^{m_{\text{out}} + \dots m_{l+2}}}_{\text{The second term in Supplementary Eq.21}} < 2^{M + \dots (L+1)m} - 1 < \underbrace{4^M - 1}_{\text{The second term in Supplementary Eq.19}}, \quad (22)$$

we see that $N_{\text{copies}}^{\text{BP}} < N_{\text{copies}}$, so the quantum BP algorithm is more efficient in training DQNNs. Such DQNNs with narrow hidden layers can be used as quantum autoencoders to compress and denoise quantum data [9]. When the width of the hidden layers is larger than that of the output layer, i.e., $m_l > m_{\text{out}}$ for $l = 1, 2, \dots, L$, we have $N_{\text{copies}}^{\text{BP}} > N_{\text{copies}}$. In this case, applying the quantum BP algorithm will require more copies of training data.

With the current methodology, the required number of copies for each training data scales exponentially with the number of qubits in the hidden and output layers. This is because an exponential number of measurements are required for quantum state tomography and the realization of backward channels. With the quantum BP algorithm, training DQNNs can be more efficient in some cases. In the future, we expect a more efficient proposal to experimentally realize the backward channel, which will improve the efficiency of the quantum BP algorithm further.

SUPPLEMENTARY NOTE 3: NUMERICAL RESULTS FOR SEVERAL MACHINE LEARNING TASKS

In this section, we classically simulate the training of DQNNs by realizing the forward channels and the backward channels without considering any experimental imperfections, and present additional numerical results.

Task: learning a two-qubit quantum channel. Here, we choose DQNN_1 mentioned in the main text to learn a two-qubit target quantum channel. The training dataset is the same as that in the main text. We numerically train DQNN_1 with 50 different initial parameters and show our numerical results in Supplementary Fig. 2. We observe that DQNN_1 shows high convergence performance, with the average converged mean fidelity above 98%.

We choose one learning curve (marked in triangles in Supplementary Fig. 2) to test the learning performance of DQNN_1. We refer DQNN_1 with parameters corresponding to the ending (starting) iteration of this training curve as the trained (untrained) DQNN_1, and then use 100 different input quantum states to test the fidelities between their corresponding output states and the desired output states given by the target quantum channel. As shown in the lower inset of Supplementary Fig. 2, for the trained DQNN_1, the mean fidelity exceeds 0.97, which separates away from the distribution of the results of the untrained DQNN_1. This contrast indicates a satisfying performance of DQNN_1.

Task: learning the ground state of molecular hydrogen (H_2). We also use DQNN_1 to learn the ground state energy of the molecular hydrogen Hamiltonian. We choose 50 different initial parameters and classically simulate the training process as presented in the main text. The results are shown in Supplementary Fig. 3. We observe that DQNN_1 converges quickly, and the average of the mean ansatz energy estimate reaches -1.826 (hartree) when excluding two abnormal instances with local minima, which is very close to the theoretical value -1.85 (hartree). This indicates the successful application of our model.

Task: learning a one-qubit quantum channel. We choose DQNN_2 mentioned in the main text to learn a one-qubit target quantum channel. In our simulation, the training dataset is the same as in the main text. Our numerical results for 50 different initial parameters are summarized in Supplementary Fig. 4. We observe that DQNN_2 shows high convergence performance in the training process, with the average converged mean fidelity above 99.5%. We also choose one of these learning curves (marked in triangles in Supplementary Fig. 4), and refer DQNN_2 with parameters corresponding to the ending (starting) iteration of the training curve as the trained (untrained) DQNN_2. We then use 100 different input quantum states to test the fidelities between their corresponding output states and the desired output states given by the target quantum channel. As shown in the lower inset of Supplementary Fig. 4, for the trained DQNN_1, the mean fidelity exceeds 0.999, which separates away from the distribution of the untrained DQNN_2 with the mean fidelity below 0.4. This contrast indicates a satisfying performance of DQNN_2.

SUPPLEMENTARY NOTE 4: EXPERIMENTAL IMPLEMENTATION OF THE DQNN

Characterization of the quantum processor

Our experiment is performed on a six-qubit superconducting quantum processor. As shown in Fig. 1(d) in the main text, the layout of qubits is carefully optimized to be a layer-by-layer structure. We denote these qubits as Q_j, where $j = 1, 2, ..., 6$, and the labels are the same as those figures in the main text. The detailed experimental wiring of qubit control lines and

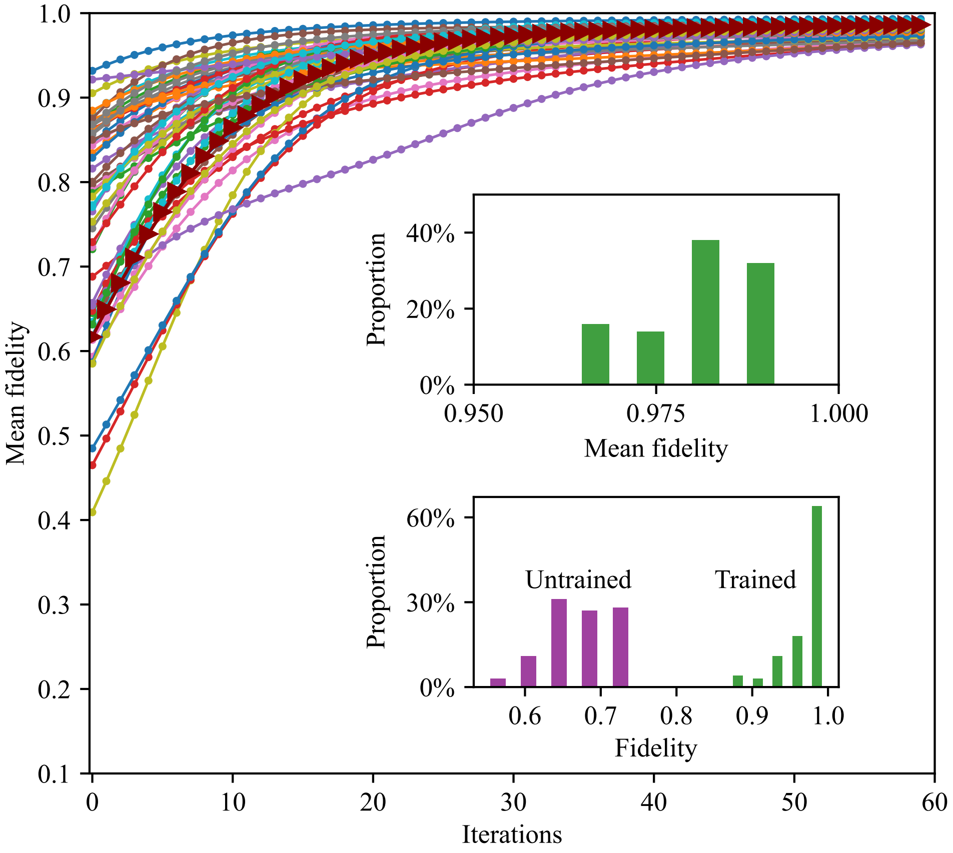

Supplementary Figure 2. **Numerical results for learning a two-qubit quantum channel.** The numerical results for training DQNN_1 with 50 different initial parameters. We plot the mean fidelity as a function of the training iterations. The upper inset shows the distribution of the converged mean fidelities for these 50 different initial parameters. We choose one of the learning curves (marked with triangles), and then randomly generate 100 different input quantum states to test the fidelities between their output states given by the target quantum channel and the trained (untrained) DQNN_1. The results are displayed in the lower inset showing the distribution of the fidelities for the trained (untrained) DQNN_1.

measurement lines are shown in Supplementary Fig. 5. We summarize the characteristic parameters of our quantum processor in Supplementary Table 1.

Synthesize the microwave control signals

Timing and microwave switch control. We note that the microwave control signals for the single-qubit gates of Q_1, Q_2, Q_5 and Q_3, Q_4, Q_6 are directly generated by the two DAC channels of a Tektronix AWG70002A (sampling rate 25 Gs per second), respectively. The single-qubit gates are implemented by 40 ns pulses with Gaussian envelopes. The tunability of the parameter θ in the DQNN is experimentally realized with a linear map between θ and the pulse amplitude. The individual addressing of each qubit is realized with the time domain separation of the microwave drives. In order to minimize the off-resonance crosstalk coming from the signal multiplexing, we add a fast microwave switch (activation time $<$ 10 ns, on-off ratio $>$ 40 dB) to each input XY control line, and turn on the switches only when the single-qubit gates are needed to be applied to the specific qubits.

Implementation of two-qubit gates with flux modulation. To realize the controlled-Phase gate in a quantum perceptron, we adiabatically tune one of the qubit frequency to bring the $|ee\rangle$ state of the control and the target qubits into resonance with the $|gf\rangle$ state . Then the two-qubit state undergoes a periodic evolution path based on the coupling Hamiltonian of the two qubits, leaving the population of the $|ee\rangle$ state intact, but a conditional geometric phase being accumulated in the $|ee\rangle$ state. Such an operation is realized with a fast step pulse of the external current threading the junction loop of the qubit to modify its frequency [10].

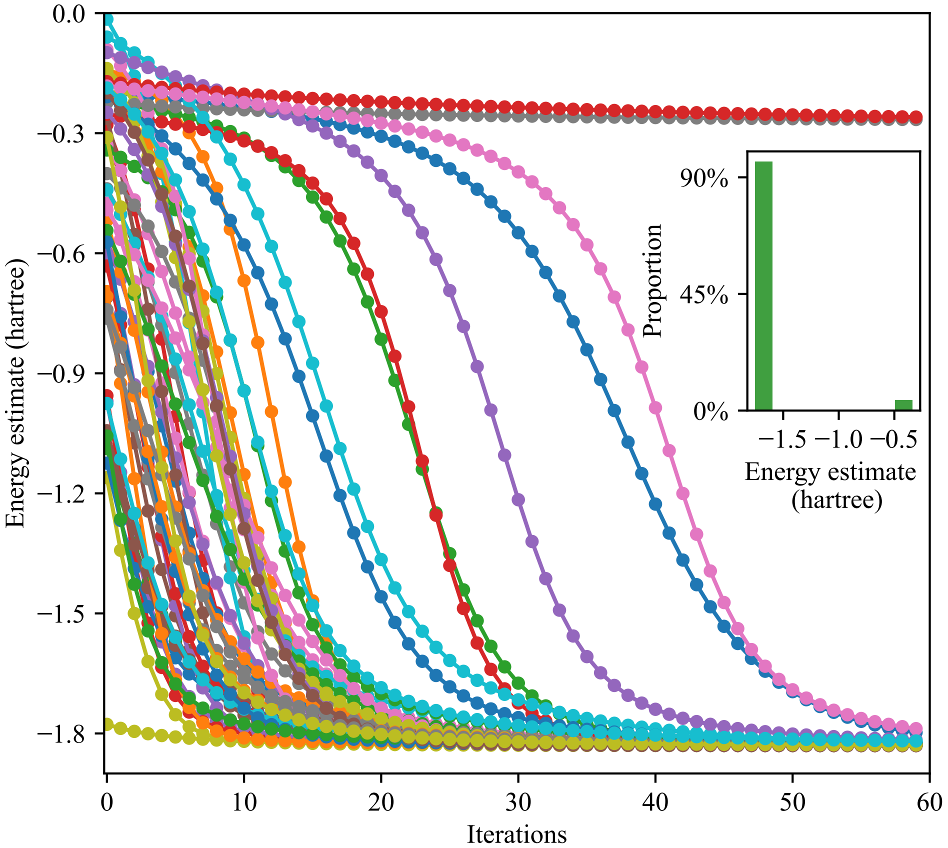

Supplementary Figure 3. **Numerical results for learning the ground state energy of molecular hydrogen.** Energy estimate as a function of the training iterations for 50 different initial parameters. The distribution of their converged energy estimates is displayed in the inset.

However, the limited bandwidth of the electronics as well as parasitic capacitances and inductances in the wiring cables lead to the distortion of the step pulses and thus the degradation of the gate performances. We adapt the method in Ref. [11] to mitigate this problem with the flux pulse compensation. We model the AWG response and the on-chip responses to the step pulse as low-pass filters, and apply real-time predistortions to the step pulse for compensations. The height and duration of the step pulse are optimized to minimize the errors in the swap process between $|ee\rangle$ and $|gf\rangle$. The comparison between the compensated and the uncompensated flux pulses is shown in Supplementary Fig. 6. We note that the optimized gate parameters do not necessarily lead to a conditional π phase, therefore, we just record the conditional phase ϕ and use it in the two-qubit gate in the perceptron. Moreover, the flux pulse of the target qubit will generally lead to the magnetic flux change not only in the target qubit loop, but also in other qubit loops, which causes the frequency and phase change of other qubit states. We calibrate the single-qubit phase of each qubit during the flux pulse through quantum state tomography, and compensate the flux-induced single-qubit phase in software by a phase shift of the following driving pulses.

Different working frequencies and phase compensation due to reference frame change. In our experiment, since the two-qubit gate requires the frequency modulation of the qubit, it is possible that the energy level resonances other than the wanted $|ee\rangle$ and $|gf\rangle$ hybridization could occur during the modulation process. Such unwanted resonances will lead to undesired state swapping that degrades the gate fidelity. In order to avoid the unwanted frequency resonances, we have set the working frequencies of the six qubits to several different configurations when executing different perceptrons in DQNN_1 and DQNN_2 (see Supplementary Table 2 for details). Meanwhile, the frequency changes of the qubits require additional time-dependent phase compensation. As illustrated in Supplementary Fig. 7(a), we calibrate the time-dependent phase change of each qubit by preparing the state $(|g\rangle+|e\rangle)/\sqrt{2}$ with a microwave driving frequency f_A when the qubit frequency is also at f_A, and then apply a predistorted step pulse in the flux control line to shift the qubit frequency to f_B. A quantum state tomography of the qubit with a driving frequency f_B is performed to extract the phase accumulation caused by the frequency change. Here we fix the time interval between the tomography pulse and the state preparation pulse, and vary the time t_{shift} between the state preparation pulse and the step pulse to calibrate the phase accumulation with t_{shift}. The calibration result is shown in Supplementary Fig. 7(b). Such a phase accumulation is also corrected in software by shifting the phases of the driving pulses after the frequency change.

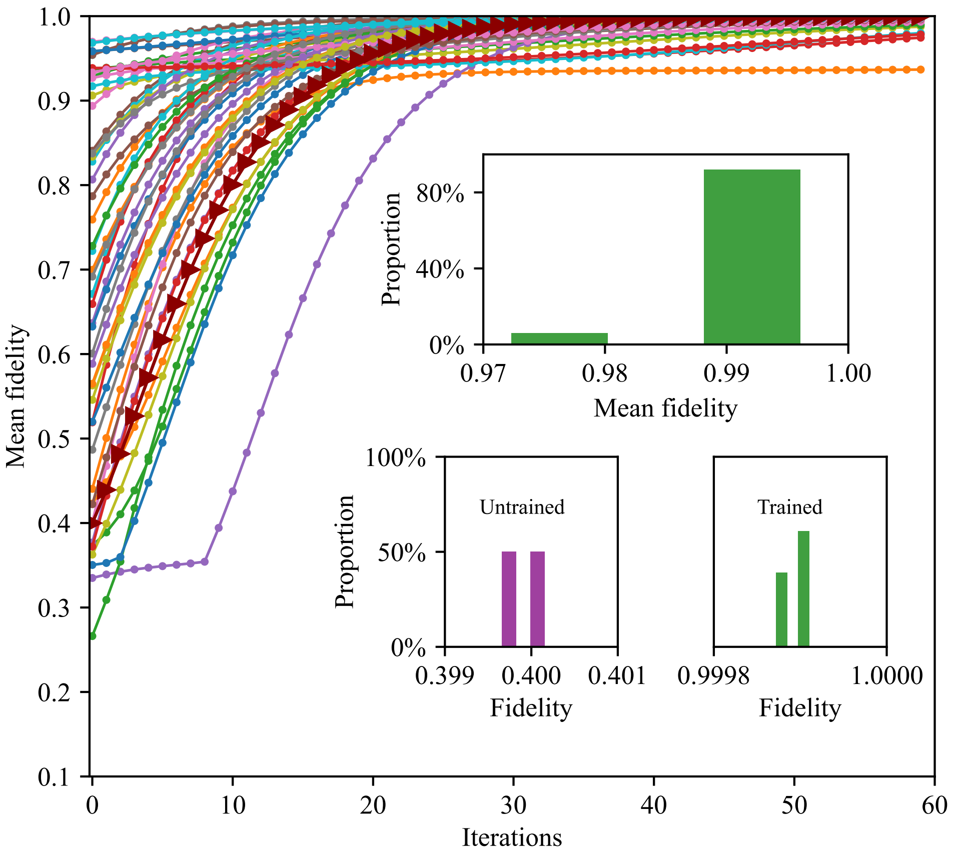

Supplementary Figure 4. **Numerical results for learning a one-qubit quantum channel.** The mean fidelity is plotted as the function of the training iterations for 50 different initial parameters. The distribution of their converged mean fidelities is displayed in the upper inset. We choose one of the learning curves (marked with triangles), then randomly generate 100 single-qubit states, separately produce their output states given by the target quantum channel and the well-trained (untrained) DQNN_2, and finally evaluate the corresponding fidelities between them. The distributions of the fidelities are shown in the lower inset.

Quantum state tomography

We extract the quantum state ρ^l of the qubits in each layer of the DQNN by carrying out the quantum state tomography. To reconstruct a single-qubit state, we perform single-qubit Pauli measurements on four bases $\mathcal{S}_1 = \{|g\rangle, |e\rangle, |+\rangle, |i\rangle\}$. To reconstruct a two-qubit state, we perform two-qubit Pauli measurements on 16 bases $\mathcal{S}_2 = \{|v_1\rangle \otimes |v_2\rangle; v_1, v_2 \in \mathcal{S}_1\}$. In our experiment, we repeat the measurement in each basis 10^4 times to obtain a probability distribution $\vec{r}$ on the two and four computational bases for the single-qubit and two-qubit cases, respectively. $\vec{r}$ is sent to a classical convex optimizer to find the density matrix ρ^l that produces the distribution as close as $\vec{r}$.

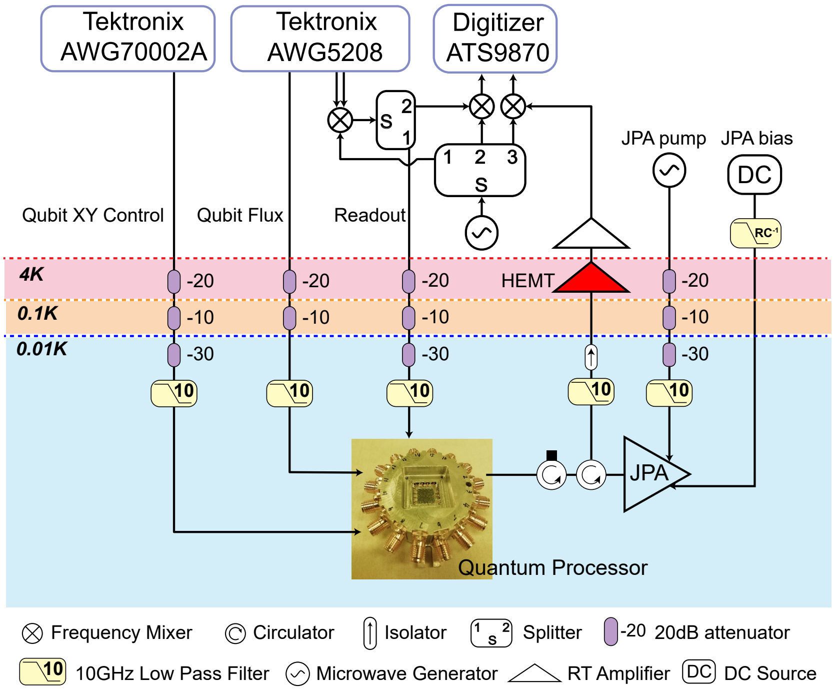

Supplementary Figure 5. **The experimental wiring of qubit control lines and measurement lines.** We plot one of two identical qubit XY control lines and one of six identical qubit flux lines for simplification purpose.

Parameters / Qubit	Q_1	Q_2	Q_3	Q_4	Q_5	Q_6
Qubit working frquency $f_{\rm Q}$ (GHz)	6.413	6.363	6.328	6.453	6.083	6.191
Qubit energy relaxation time T_1 (μs)	4.2	6.1	5.1	8.2	10.0	10.6
Qubit Ramsey dephasing time T_2 (μs)	2.2	1.9	4.8	8.4	18.2	11.8
Qubit anharmonicity $E_{\rm C}/2\pi$ (MHz)	194	217	206	196	207	208
Readout resonator frequency $f_{\rm R}$ (GHz)	7.10	7.16	7.13	7.22	7.12	7.21
Qubit-readout-resonator coupling strength $g_{\rm QR}/2\pi$ (MHz)	69	72	81	66	78	65
Qubit-bus-resonator1 coupling strength $g_{\rm QB1}/2\pi$ (MHz)	0	0	36	35	37	34
Qubit-bus-resonator2 coupling strength $g_{\rm QB2}/2\pi$ (MHz)	32	31	34	32	0	0
Internal quality factor of the readout resonator $Q_{\rm I,R}\,(10^3)$	102	83	130	15	85	92
Coupled quality factor of the readout resonator $Q_{\rm C,R}\,(10^3)$	20	15	20	7.6	7.4	8.0

Supplementary Table 1. **Characteristic parameters of the quantum processor.**

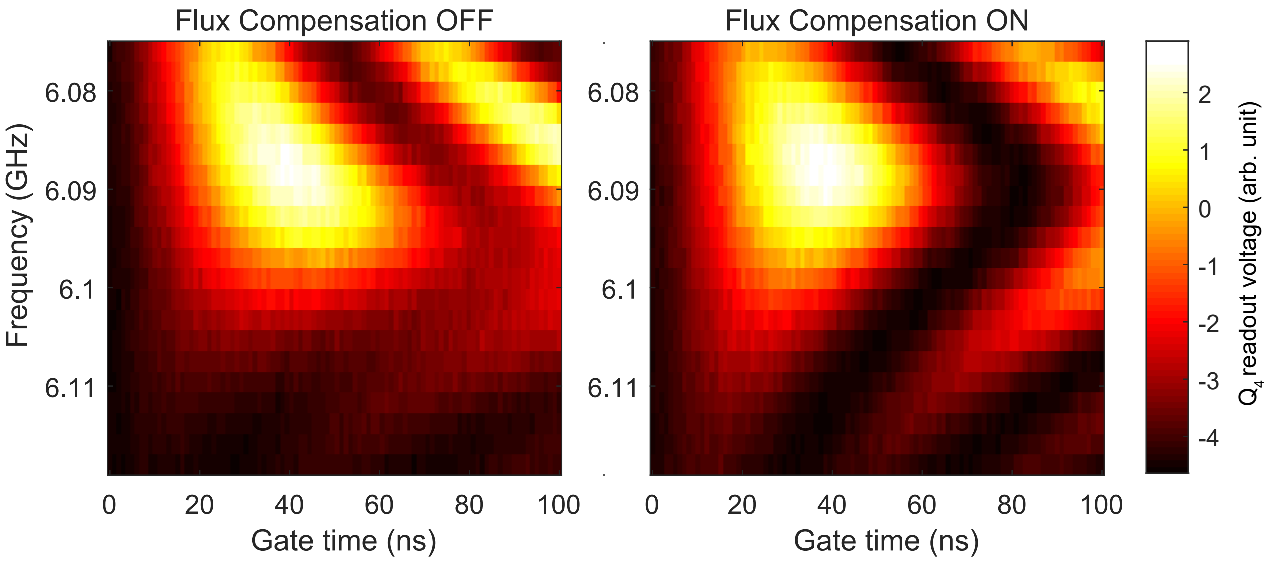

Supplementary Figure 6. **The comparison between the swap operations with and without the flux compensation.** With the frequencies of all other qubits well below 5.8 GHz, we prepare Q_4 and Q_5 in the $|eg\rangle$ state, and take a step pulse in the flux line to modulate the frequency of Q_4 down to reach the resonance with the $|ge\rangle$ state. The modulated qubit frequency and the duration of the step pulse are varied in the experiment. Compared with the uncompensated step pulse, the predistortion compensation method has successfully recovered the chevron pattern of the expected $|ge\rangle$ and $|eg\rangle$ swap process.

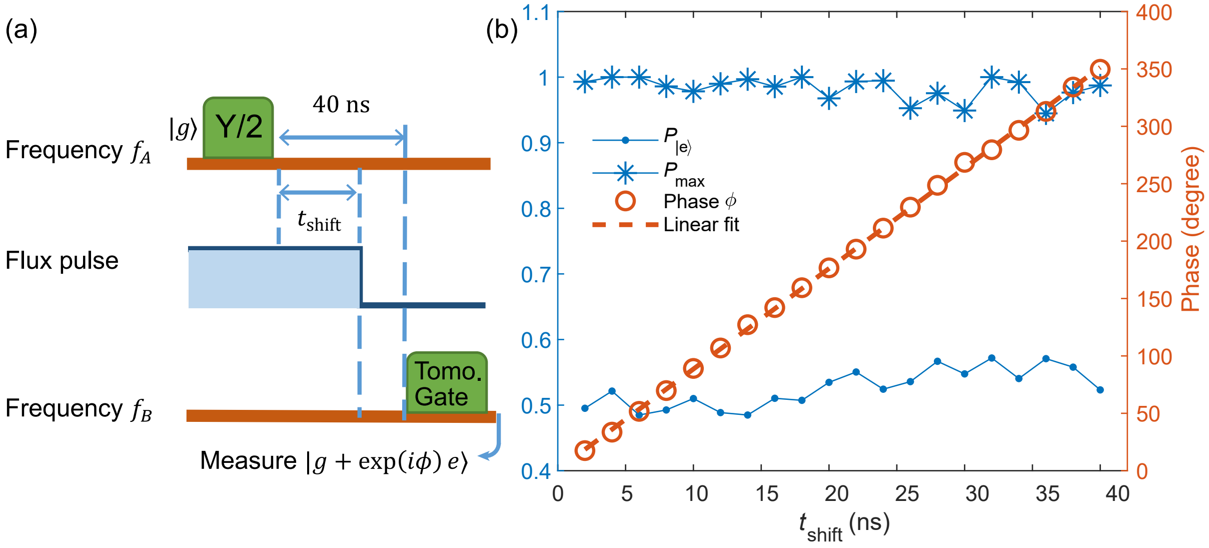

Supplementary Figure 7. **Calibration of the single-qubit phase induced by the shift of the working frequency.** (a) The experimental pulse sequence. (b) The experimental result for the frequency shift process of Q_4. The blue dots denote the probability of measuring $|e\rangle$ state $P_{|e\rangle}$. The blue star marks denote P_{max}, which is the larger eigenvalue of the single-qubit density matrix. The near-unity values of P_{max} indicate the measured final quantum states are close to pure states. The red circles denote the phase ϕ extracted from the final quantum states in the form $|g\rangle + e^{i\phi}|e\rangle$. The dashed line is a linear fit to the red circles to infer the desired frequency shift.

DQNN$_1$

Percep	Qubits	Rotation angle	Q_1	Q_2	Q_3	Q_4	Q_5	Q_6	Time	Phase	Fidelity
$U_{1,1}^1$	Q_1, Q_3	$4.75, -1.91$	6.413	6.364	6.320	6.453	< 5.8	< 5.8	62	$175°$	0.986
$U_{2,1}^1$	Q_2, Q_3	$-1.46, 6.14$							52	$180°$	0.981
$U_{1,2}^1$	Q_1, Q_4	$-3.61, -0.97$							92	$180°$	0.978
$U_{2,2}^1$	Q_2, Q_4	$4.56, 1.60$							82	$-155°$	0.981
$U_{1,2}^2$	Q_3, Q_6	$-2.05, -0.40$	< 5.8	< 5.8	6.328	6.453	6.08	6.191	64	$176°$	0.988
$U_{1,1}^2$	Q_3, Q_5	$6.03, -2.32$							64	$-117°$	0.972
$U_{2,2}^2$	Q_4, Q_6	$-3.47, -0.31$			< 5.8				64	$-157°$	0.990
$U_{2,1}^2$	Q_4, Q_5	$-1.71, 0.92$							60	$-165°$	0.982

DQNN$_2$

Percep	Qubits	Rotation angle	Q_1	Q_2	Q_3	Q_4	Q_5	Q_6	Time	Phase	Fidelity
$U_{1,1}^1$	Q_1, Q_3	$3.32, -1.46$	6.413	6.364	6.320	6.453	< 5.8	< 5.8	62	$175°$	0.986
$U_{1,1}^2$	Q_2, Q_3	$6.10, -6.23$							52	$180°$	0.981
$U_{1,1}^3$	Q_2, Q_4	$-1.05, 0.34$							82	$-155°$	0.981
$U_{1,1}^4$	Q_4, Q_6	$6.22, -5.43$	<5.8	<5.8	< 5.8	6.453	6.08	6.191	64	$-157°$	0.990
$U_{1,1}^5$	Q_5, Q_6	$2.57, 5.56$							60	$-130°$	0.985

Supplementary Table 2. **Experimental parameters for training DQNNs.** In our experiments, DQNN$_1$ (DQNN$_2$) denotes the three-layer (six-layer) DQNN. The quantum perceptrons are applied in the order from the top to the bottom in the first column when running the DQNN. $U_{(i,j)}^l$ denotes the quantum perceptron which acts on the i-th qubit at layer $l-1$ and the j-th qubit at layer l. Each perceptron acts on the qubits listed in the second column. When applying different perceptrons, we need to set the qubits to different frequencies (in GHz) as listed in the fourth to ninth columns. We also show the operation time (in ns) and the rotation angle for the controlled-Phase gate in each perceptron. The experimentally characterized two-qubit gate fidelity for the controlled-Phase gate in each perceptron is displayed in the last column. The target quantum channels are constructed using the DQNN ansatz with randomly chosen single-qubit rotation angles of each quantum perceptron, which is shown in the third column (in rad).

* These two authors contributed equally to this work.
† E-mail: dldeng@tsinghua.edu.cn
‡ E-mail: luyansun@tsinghua.edu.cn

[1] J. Biamonte, P. Wittek, N. Pancotti, P. Rebentrost, N. Wiebe, and S. Lloyd, Quantum machine learning, Nature **549**, 195 (2017).
[2] V. Dunjko and H. J. Briegel, Machine learning & artificial intelligence in the quantum domain: A review of recent progress, Rep. Prog. Phys. **81**, 074001 (2018).
[3] S. Das Sarma, D.-L. Deng, and L.-M. Duan, Machine learning meets quantum physics, Phys. Today **72**, 48 (2019).
[4] M. Cerezo, G. Verdon, H.-Y. Huang, L. Cincio, and P. J. Coles, Challenges and opportunities in quantum machine learning, Nat. Comput. Sci. **2**, 567 (2022).
[5] A. Dawid, J. Arnold, B. Requena, A. Gresch, M. Płodzień, K. Donatella, K. A. Nicoli, P. Stornati, R. Koch, M. Büttner, *et al.*, Modern applications of machine learning in quantum sciences, arXiv:2204.04198 (2022).
[6] K. Beer, D. Bondarenko, T. Farrelly, T. J. Osborne, R. Salzmann, D. Scheiermann, and R. Wolf, Training deep quantum neural networks, Nat. Commun. **11**, 808 (2020).
[7] K. Beer, Quantum neural networks, arXiv:2205.08154 (2022).
[8] P. Chamorro-Posada and J. C. Garcia-Escartin, The SWITCH test for discriminating quantum evolutions, arXiv:1706.06564 (2023).
[9] D. Bondarenko and P. Feldmann, Quantum Autoencoders to Denoise Quantum Data, Phys. Rev. Lett. **124**, 130502 (2020).
[10] R. Barends, C. M. Quintana, A. G. Petukhov, Y. Chen, D. Kafri, K. Kechedzhi, R. Collins, O. Naaman, S. Boixo, F. Arute, *et al.*, Diabatic gates for frequency-tunable superconducting qubits, Phys. Rev. Lett. **123**, 210501 (2019).
[11] M. A. Rol, L. Ciorciaro, F. K. Malinowski, B. M. Tarasinski, R. E. Sagastizabal, C. C. Bultink, Y. Salathe, N. Haandbaek, J. Sedivy, and L. DiCarlo, Time-domain characterization and correction of on-chip distortion of control pulses in a quantum processor, Appl. Phys. Lett. **116**, 054001 (2020).
